# Mental Burden of Hospital Workers During the COVID-19 Crisis: A Quanti-Qualitative Analysis

**DOI:** 10.3389/fpsyt.2021.622098

**Published:** 2021-04-23

**Authors:** Amandine Luquiens, Jennifer Morales, Marion Bonneville, Hugo Potier, Pascal Perney, Gilles Faure, Astrid Canaguier

**Affiliations:** ^1^Addictions Department, CHU Nîmes, Univ Montpellier, Nîmes, France; ^2^COVIPSY unit, CHU Nîmes, Univ Montpellier, Nîmes, France; ^3^Paris-Saclay University, Univ. Paris-Sud, UVSQ, CESP, INSERM, Villejuif, France; ^4^CUMP, Mas Careiron, France; ^5^BESPIM, CHU Nîmes, Univ Montpellier, Nîmes, France; ^6^SAMU, CUMP, CHU Nîmes, Univ Montpellier, Nîmes, France

**Keywords:** mental burden, hospital workers, COVID−19, volonteers, Qualitative–quantitative analysis

## Abstract

**Context:** This study is a quanti-qualitative analysis of all contacts to a helpline service for hospital workers during the COVID-19 crisis. Our aim was to describe the nature of mental burden in hospital workers and factors subjectively associated to this burden from the workers' perspective.

**Methods:** We included all 50 contacts from 25 different workers and 10 different professions over the course of 1 month. We described the corpus and reported the computerized qualitative analysis of summary of contacts. We performed a descendant hierarchical analysis and analyzed specificities of classes of age with a correspondence factor analysis.

**Results:** The corpus was composed of three classes: (1) distress specific to the COVID-19 situation, (2) help provided, and (3) pre-existing psychological vulnerability. Factors subjectively responsible for mental distress were: (a) the contamination risk, (b) confinement, and (c) the rapidly evolving situation and changing instructions. Lack of “COVID-free time” seemed to increase negative emotions. Reassignment to a high viral density unit was a stressor, especially in older workers. Young workers mentioned pre-existing vulnerability more than others. Fear of death was shared by all classes of age, regardless of the objective risk of contamination.

**Discussion:** Hospital workers experience mental distress factors both in common with the general population and specific to the hospital environment. Preserving and organizing support for the mental health of all hospital workers is a critical challenge, including those with poorly recognized professions. Leads for institutions to avoid additional stressors for hospital workers are presented. Young workers with pre-existing vulnerability seem particularly impacted.

## Highlights

- The qualitative nature of the study allows to describe the content of mental burden linked to COVID crisis and factors subjectively identified by hospital workers as responsible for it.
- Hospital workers cumulate stressors: (a) the contamination risk (b) confinement (c) rapidly changing instructions (d) reassignment to high viral density unit.
- Young workers mentioned pre-existing vulnerability more than others.
- Fear of death was shared by all classes of age disregarding the objective risk of contamination.

## Introduction

The mental distress linked to the COVID-19 crisis is health-related, but is also due to confinement, and these two issues have been highlighted in scientific literature (1). The most common difficulties in the general population appear to be sleep disorders, anxiety, and depression (2), that can in some case meet a high level of distress (3). Health care providers experience additional stressors (4), with at least one third of them suffering from insomnia. Several factors have been reported to be associated with the mental burden of the crisis in health care providers: level of education, working in an isolation unit, and fear of being contaminated (4). Most of the data came from large surveys among self-selected participants. To our knowledge, very few qualitative studies has been published to explore qualitatively the nature of the mental burden of the crisis among help-seeking professionals (5–7). The relevance of explorative qualitative research has, however, been highlighted by some authors (8). The authors reported the growth of negative emotions, including fatigue and anxiety for one-self and for one's loved ones, but also coping strategies and positive emotions linked to self-accomplishment in work. No qualitative study explored the mental burden of the COVID crisis in other hospital workers, either in other care workers (e.g., nurse assistants or medical doctors) or in cleaning, catering, and administrative staff working in the hospital. Some factors were reported to affect professional mental burden: exposure to numerous COVID patients and frontline workers, being a nurse, being a woman, having children among household members, and moderate to low knowledge of the infection (9–11). The age of health care providers could also influence the level of severity of mental distress in this population. Older people are more at risk of severe forms of COVID and death if contaminated (12). They also seem to be more vulnerable to post-traumatic stress symptoms (13). On the other side, young people in the general population have been reported to have around a 40% tendency to experience psychological problems in this context (3, 14). People under 35 could be more impacted (2). Young women could endure the heaviest burden (15). However, no qualitative work has been reported to distinguish the nature of mental distress in relation to age. Recent cross-sectional quantitative studies can hardly respond to causal factors associated with mental burden. We conducted a quanti-qualitative analysis of all contacts to a local helpline service organized by a French hospital to respond to the mental distress of hospital workers, over the period of 1 month. Our aim was to describe the nature of mental burden in hospital workers, including poorly recognized professions, and factors subjectively associated with this burden from the workers' perspective.

## Methods

### Population

An institutional helpline was organized by the Universitary Hospital of Nîmes, France, 1 week after the beginning of confinement in the context of the COVID-19 crisis. The helpline aimed to provide psychological support for all local hospital workers, including health care providers, but also people handling logistical or administrative work. Helpers were volunteer psychologists and psychiatrists. A communication to inform all hospital workers of the helpline number was realized by recurrent mailing, a poster campaign, and informal communication. We report the analysis of all contacts between the creation of the helpline on the 25th of March, 2020, and the 27th of April 2020. Workers could call the helpline several times and/or have a face-to-face interview if needed.

### Data Collected

Basic sociodemographic (age and sex) and professional characteristics of callers were collected: work with patient or logistic/administrative work, contact with patients infected by SARS-Cov 2 (yes/no/na, i.e., no contact with patient) (10), and reassignment to a high viral density department (yes/no). For each contact, helpers wrote a summary and quote, in accordance with his/her clinical judgment, the severity of anxiety, the thymic state, and a global assessment of psychological current distress on five- point Likert scales. Presence of the following difficulties was collected in binary variables according to the clinician judgment: sleep difficulties, feelings of guilt, and feelings of exhaustion. The data were routinely and prospectively registered in the RedCap system. Data collection was anonymous. Contactors were systematically orally informed of the informatic data collection. We retrospectively analyzed the collected data, respecting the MROO3 methodology legal framework.

### Data Analysis

The summary of contacts written by the helpers were merged to create a corpus. A content analysis was performed on the corpus, facilitated by the software Iramuteq 0.7 alpha 2., a textual data analysis. Given the emotional impact of the still current crisis, we chose to use a computerized analysis to prevent as much as possible any *a priori* effect on the nature of the psychological burden linked to the crisis among hospital workers. We described the corpus after a lemmatization phase. Lemmatization allows reduction of words to their radicals, called a reduced form and classified as “analyzable” (nouns, verbs, adjectives, adverbs) or “supplementary” (prepositions, pronouns, verb to be or to have, etc.) forms. We chose to keep in the analysis only analyzable forms. Frequency of forms was calculated for the whole corpus and a word-cloud was generated. We conducted a lexical analysis using the descending hierarchical classification method “Reinart” to obtain stable classes of forms that were the most significant structures of the corpus. The Reinart method was conducted as follows: (i) Text segmentation into elementary context units (ECUs); (ii) Definition of a contingency table of “analyzable” reduced forms occurring at least four times in the corpus called “analyzed forms” and elementary context units (ECUs); and (iii) Top-down hierarchical classification with a double classification of text segment groupings. In each obtained stable class were then listed the most character-defining forms, in their presence or their absence (chi-square), comparatively to their presence in the whole corpus (tested by chi-square). A dendrogram was generated. Then, we qualitatively analyzed theses classes in the light of the context in which the forms were used in the text. Furthermore, an analysis on age class [30 or less (*n* = 6), 30 to 50 (*n* = 11), 50 years old or more (*n* = 8)] was conducted to identify any specificity linked to age in the corpus. Relative frequency and a correspondence factor analysis were performed to identify the significant forms from each class of age.

### Ethics

Data collection was anonymous. We retrospectively analyzed the collected data. Contactors were systematically orally informed of the informatic data collection. We obtained approval from the local Institutional Review Board n° 20.09.06.

## Results

### Sample

The study reports the whole activity of the helpline unit: 25 different hospital workers contacted the helpline, leading to 50 contacts. The number of contacts by workers ranged from 1 to 7. The workers were seven nurses, one nurse manager, one nurse student, four nurse assistants, two catering staff people, one secretary, four cleaning people, three radio manipulators, one lab technician, and two people working in a geriatric center with unspecified positions. Table 1 reports the sample characteristics of workers and contacts. All workers except one were women. Age range was 20–62 years old: six workers were 30 or less, eleven were 30- to 50-years-old, and eight 50-years-old or more. Most workers were health providers (84%), and more than half were in contact with COVID patients (56%). The mean global psychological burden as assessed by the clinician helpers was moderate (3.11 on 5). Sleep disturbance was reported in half of contacts. Exhaustion and feelings of guilt were reported in more than a quarter of contacts (both 26%).

**Table 1:** Characteristics of the sample.

	**Mean**	**sd**
Sex (female) (*n* = 25) (*n*, %)	24 (96%)	
Age (*n* = 25)	44.16	11.45
Worker with patient vs. logistic/administrative work (*n* = 25) (*n*, %)	21 (84%)	
Worker with COVID patient (yes) (*n* = 25) (*n*, %)	14 (56%)	
Reassigned worker (yes) (*n* = 25)	4 (16%)	
Psychological assessment of each contact (*n* = 50):		
Level of anxiety (0 to 4) (*n* = 46)	3.15	1.01
Mood state (3= neutral) (*n* = 46)	2.35	0.77
Sleep disturbance (yes) (*n*, %)	25 (50%)	
Exhaustion (yes) (*n*, %)	13 (26%)	
Feeling of culpability (yes) (*n*, %)	13 (26%)	
Global assessment of psychological burden (*n* = 46)	3.11	0.80

### Statistics of the Corpus

**Figure 1:**
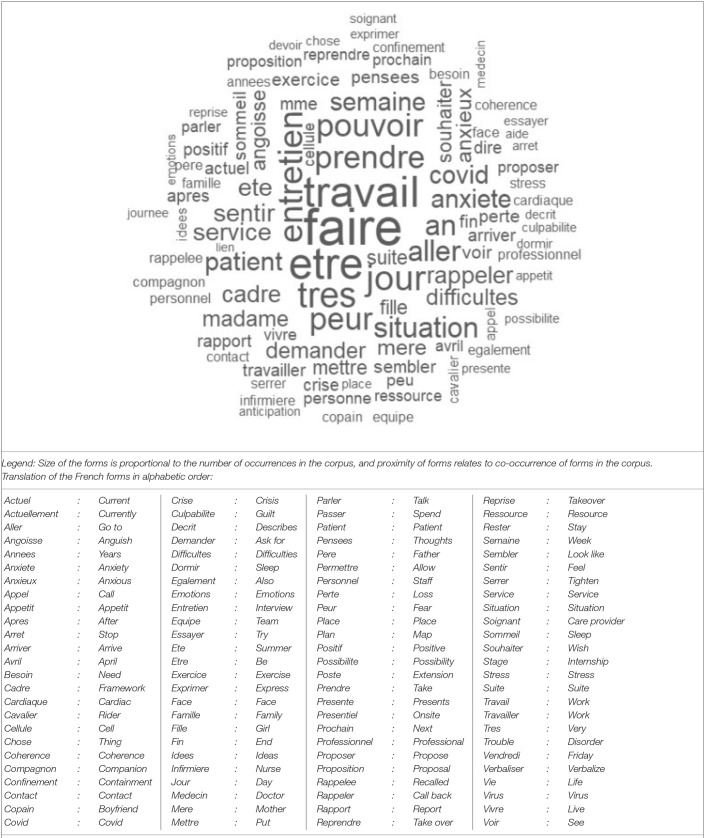
Word cloud representing the relative importance and co-occurrence of the 100 main forms in the corpus.

We count 7,827 occurrences in the corpus, of which 1,325 were active forms. The different active forms with a frequency ≥ 3 are 350. Figure 1 represents the relative importance and co-occurrence of the 100 main forms in the corpus.

As represented in Figure 1, the forms with 20 or more occurrences in the corpus are “to be, work, very, day, interview, take, fear, go to, situation, patient, week, covid, anxiety, department, feel, summer.” Taken together, all French forms referring to an anxious state (i.e., “angoissé, angoisse, anxiété, anxieux”) occur 60 times in the corpus and appear to be the main complaint of the hospital workers. The forms “sommeil, dormir” (i.e., “sleep/to sleep”) appear 22 times in the corpus.

In the light of the context of use in the corpus, the form “situation” appears to be a portmanteau word referring to a broad panel of current professional and domestic threats (e.g., risk of contamination or lack of materials) and changes in work organization due to the COVID crisis, as well as the confinement situation. Some workers refer to “the whole situation,” or “the current situation.” The following quotation illustrates this: “the containment situation, changes in the workplace: new nursing staff, new agents, new staff coming from outside the hospital.” Forms “go to” and “work” are often used together, either to report a difficulty in going to work, or because of some workers being on sick leave. The form “situation” also occurs in sentences reporting psychological distress or symptoms: “a nurse's assistant who takes care of a COVID patient, she has some sleeping problems and nightmares related to the current situation.” In addition to sleep difficulties, we note the following occurrences in the corpus: “fatigue” five times, “concentration” four times, “sad/sadness” nine times, “suicidal” six times, and “trauma/traumatic” eight times.

### Hierarchical Descendant Analysis Method Reinart

**Figure 2:**
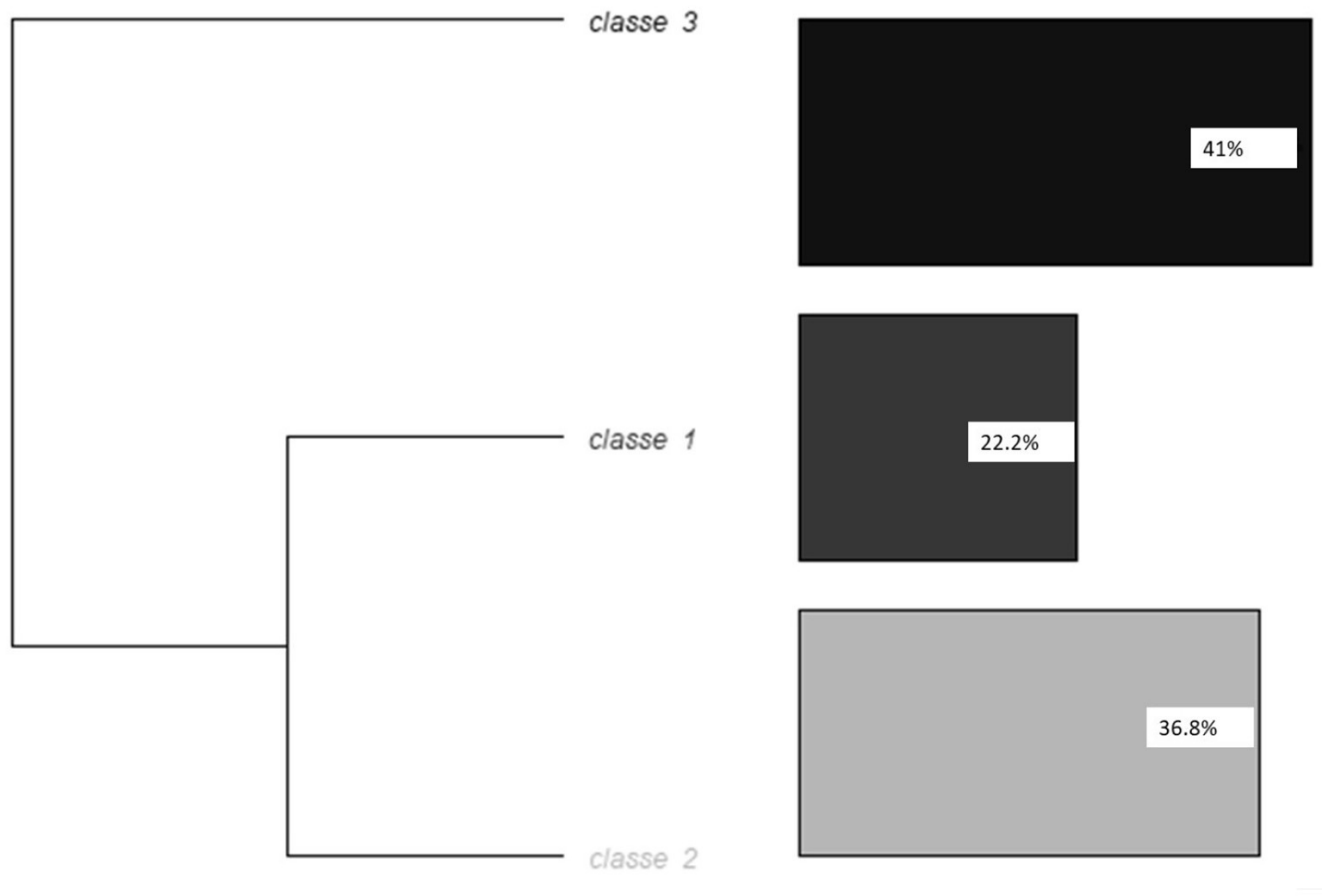
Dendrogram of the hierarchical descendant analysis method Reinart.

The corpus was automatically divided into 395 segments of text. The analysis retained three different classes; 30% of segments were classed in one of the three classes at the end of the analysis. Figure 2 represents the dendrogram of the hierarchical descendant analysis with the Reinart method.

Class 1 corresponds to the mental burden of the COVID crisis in hospital workers. The forms significantly associated with class 1 were: something (<0.0001), fear (0.00014), work (0.00717), feel (0.00717), hold out (0.00977), and talk (0.03782). The variables significantly associated with class 1 were: being middle aged (0.01590), being reassigned (0.00080), being in contact with COVID patients (<0.0001), and with being in contact with patients vs. administrative or logistic work (0.01368). However, care workers were not the only ones to report fear: “a lot of anxiety related to the COVID [...]. She explains that she wants to take it upon herself and not express her anxiety to her relatives in order not to add to their worries,” said a cleaning woman. The fear appears to be related to several factors: (a) the contamination risk: “she talks about the fear of dying if she is infected with COVID due to her age and her lung problems with chronic bronchitis.” for one's self or relatives; (b) confinement itself and additional precautions some workers took to avoid contaminating their relatives, leading to isolation: “In the anxious anticipation not of contracting the disease but of dying alone.” “Anticipates also the end of the confinement and the relational difficulties which will result from it because one will be afraid.”; and (c) the rapidly evolving situation and instructions: “intolerance to the unknown, [describes] a lot of changes at work. Does not know where she is going, lack of information. “Fear appears several times to be enhanced by a kind of general panic and absence of a COVID-free space or time: “she considers that the problem is the omnipresence of the coronavirus and the related risks in the environment: radio, newspapers, work, informal team time-out.”

Class 2 corresponds to the help provided and its formal organization. The forms significantly associated with class 2 were: exercise (0.00012), April (0.00034), end (0.00073), call (0.00112), proposition (0.00271), ask (0.00558), emotions (0.00558), Friday (0.00759), breath (0.00759), confirm (0.00759), propose (0.01511), be (0.02008), patient (0.02134), seem (0.02134), helpline (0.02134), contact (0.02134), anticipation (0.02134), phone (0.02134), recontact (0.02134), Tuesday (0.02134), hotline (0.02134), wish (0.04035), and day (0.04970). The variables significantly associated with class 2 were: age ≥50 (<0.0001) and administrative or logistic work (<0.0001).

Class 3 corresponds to the pre-existing psychological vulnerability and life history. The forms significantly associated with class 3 were: mother (<0.0001), parent (0.00106), daughter (0.00256), years (0.00614), step[father] (0.00614), live (0.01317), boyfriend (0.01468), sister (0.01468), conflict (0.01468), apartment (0.01468), life-partner (0.03539), describe (0.03539), life (0.03539), child (0.03539), results (0.03539), mood (0.03539), high-school (0.03539), and year (0.01964). The variables significantly associated with class 3 were: not being reassigned (0.00088), being in contact with patients vs. administrative or logistic work (0.02236), no contact with COVID patients (0.00265), and with being under 30 (<0.0001). The following quotation illustrates the pre-existing vulnerability: “Has had a previous history of depression, dark thoughts for the past 2 years, the current situation increases her symptoms.”

### Analysis by Class of Age: Role of Age in the Mental Burden

**Figure 3:**
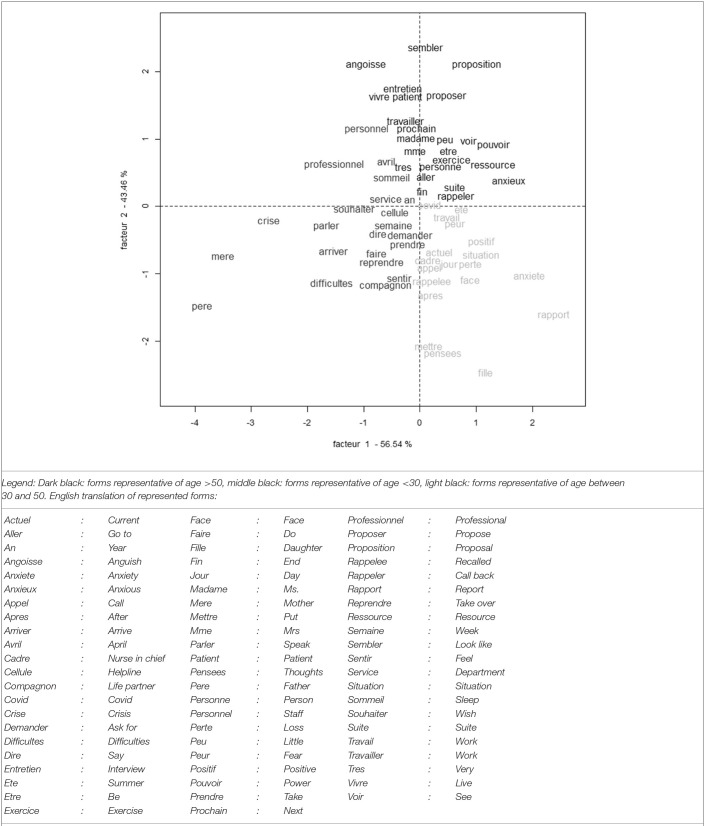
Graphical representation of two dimensions' correspondence factor analysis based on class of age and forms of each class of age with the highest relative frequency in this class.

Figure 3 represents graphically the two dimensions' correspondence factor analysis based on class of age and forms of each class of age with the highest relative frequency in this class.

The oldest class reports relatively more than the other classes the following forms “anxious, being able, work”. The forms replaced in the context of use in the corpus demonstrate an anxiety of not being capable enough or not holding out, particularly when reassigned in a more intensive care unit or a COVID unit. The following quotations illustrate that point: “this announcement [reassignment] seems to generate a lot of anxiety for different reasons. First of all due to doubts in her professional abilities: understanding the management of the care service, the management of such a big team, the specific computer tools, etc.”, “Tackles the fear of committing malpractice because she does not know the department in which she works, feeling isolated at work.” Forms reporting the intervention of the helpers are also more frequent in the class of age: “exercise, resource, interview, proposition,” indicating that this anxiety could be more susceptible to stress regulation techniques used by the helpers, or that the helpers propose more often such techniques and a follow up. The following quotation illustrates that point: “She says she feels less lonely and accepts the offer to be called back...”

The middle-aged class reports relatively more than the other classes the following forms: “daughter, fear, [COVID] positive.” The context of use in the corpus shows a fear of death of relatives and of their own death due to COVID. Fear of death in this class is reported for some workers related to exposure to TV media, and not necessarily due to fear of contamination at work: “she copes well with the current situation at her workplace in relation to COVID, [she] is anxious only when she watches the news.” The following quotations illustrate that point: “she worries about their loved ones, she is afraid that something will happen to them, afraid of dying,” “anxiety for her daughter.” Difficulties linked to confinement and living with co-confined people is also reported, particularly when young adults came back to their parents' home.

The youngest class reports relatively more than the other classes the forms “difficulties” and mother,” “father.” The form difficulties refers often to symptoms: “sleep difficulties,” “concentration difficulties,” “difficulty to breath,” but also “relational difficulties.” As mentioned above, the youngest workers showed more than others pre-existing vulnerability and familial relational difficulties linked in part to confinement. Table 2 details the highest relative frequencies in each class of age, and translation in English.

**Table 2:** Forms of each class of age with the highest relative frequency in this class, and translation in English, in the alphabetic order.

	**Aged under 30 (*n* = 6)**	**Aged between 30 and 50 (*n* = 11)**	**Aged over 50 (*n* = 8)**
Actuel : Current	11.66	14.16	8.73
Aller : Go to	20.41	19.83	26.2
An : Year	23.32	19.83	21.83
Angoisse : Anguish	17.49	0	21.83
Anxiete : Anxiety	2.92	36.83	17.47
Anxieux : Anxious	2.92	19.83	19.65
Appel : Call	8.75	11.33	6.55
Apres : After	11.66	17	6.55
Arriver : Arrive	20.41	8.5	6.55
Avril : April	11.66	5.67	10.92
Cadre : Nurse in chief	17.49	22.66	13.1
Cellule : Helpline	11.66	8.5	8.73
Compagnon : Life partner	11.66	11.33	4.37
Covid : Covid	17.49	19.83	19.65
Crise : Crisis	26.24	2.83	6.55
Demander : Ask for	17.49	17	13.1
Difficultes : Difficulties	29.15	14.16	6.55
Dire : Say	14.58	11.33	8.73
Entretien : Interview	26.24	8.5	41.48
Ete : Summer	11.66	22.66	19.65
Etre : Be	26.24	31.16	45.85
Exercice : Exercise	8.75	11.33	15.28
Face : Face	5.83	14.16	6.55
Faire : Do	61.22	42.49	28.38
Fille : Daughter	8.75	28.33	4.37
Fin : End	11.66	11.33	13.1
Jour : Day	20.41	39.66	24.02
Madame : Ms.	11.66	11.33	21.83
Mere : Mother	40.82	2.83	4.37
Mettre : Put	14.58	22.66	4.37
Mme : Mrs	11.66	8.5	15.28
Parler : Speak	17.49	5.67	6.55
Patient : Patient	17.49	8.5	30.57
Pensees : Thoughts	11.66	22.66	4.37
Pere : Father	26.24	2.83	0
Personne : Person	8.75	8.5	13.1
Personnel : Staff	11.66	2.83	10.92
Perte : Loss	5.83	19.83	10.92
Peu : Little	8.75	8.5	13.1
Peur : Fear	17.49	31.16	24.02
Positif : Positive	5.83	17	10.92
Pouvoir : Power	8.75	25.5	37.12
Prendre : Take	29.15	28.33	19.65
Prochain : Next	8.75	5.67	13.1
Professionnel : Professional	14.58	2.83	8.73
Proposer : Propose	5.83	5.67	15.28
Proposition : Proposal	2.92	5.67	17.47
Rappelee : Recalled	8.75	11.33	6.55
Rappeler : Call back	11.66	19.83	19.65
Rapport : Report	0	25.5	8.73
Reprendre : Take over	11.66	11.33	6.55
Ressource : Resource	2.92	11.33	13.1
Semaine : Week	26.24	17	15.28
Sembler : Look like	8.75	2.83	21.83
Sentir : Feel	23.32	22.66	10.92
Service : Department	23.32	14.16	17.47
Situation : Situation	11.66	33.99	19.65
Sommeil : Sleep	14.58	8.5	13.1
Souhaiter : Wish	20.41	8.5	10.92
Suite : Suite	8.75	14.16	15.28
Travail : Work	29.15	42.49	32.75
Travailler : Work	11.66	5.67	15.28
Tres : Very	32.07	19.83	32.75
Vivre : Live	11.66	2.83	15.28
Voir : See	5.83	11.33	17.47

*NB: forms represented on Figure 3*.

## Discussion

Our quanti-qualitative study on the mental burden of the COVID crisis in hospital workers supports previous studies on the general population and on health care providers on the prevalence of sleep disorders and anxiety. The qualitative nature of the study allows us to describe the content of this burden and factors subjectively identified by our population as responsible for it. Previous research demonstrated that access to a local help facility corresponds to an expectation of a broad majority of the health professionals (16).

### General Factors Related to the Mental Burden of the COVID Crisis in Hospital Workers

Our study showed that hospital workers cumulate anxiety factors: (a) factors related to the general context, as in the general population, i.e., confinement; (b) factors linked to the risk of contamination at work; and (c) factors linked to the rapidly evolving situation and changes in organization at work due to new protocols and changing instructions evolving with new scientific knowledge. This last point supports previous qualitative findings in nurses, reporting the need for good communication between managers and front-line nurses to humanize shift patterns (6).

Isolation in this context also seems particularly toxic. The role played by isolation has been reported as associated with psychological distress in the general population as well (17). Fear of dying alone illustrates this additional burden of the crisis in the population of hospital workers. The absence of a psychological airlock or “COVID-free time,” as well as the toxicity of exposure to media, has been highlighted by our sample, and supports previous findings (18, 19).

### Role of Age

Intolerance to the unknown and to rapidly evolving situations and instructions seemed to be increased in older workers who were reassigned and had to work with unknown staff in unusual functions. Our qualitative findings with no a priori assumptions could provide a way to understand the higher vulnerability to post-traumatic stress symptoms in older health professionals reported in a previous study (13); reassignment and lower flexibility in work routine could act as mediators between age and trauma. These findings should be confirmed by future quantitative research. They have direct clinical implications in terms of management to preserve well-being among older workers during a rapidly evolving crisis; reassignment should be accompanied and the difficulty in this class of adapting to to a new environment, new tools, and colleagues should be taken into account. On the other hand, pre-existing vulnerability was obvious for the youngest workers, and could explain the higher impact previously described in young health care providers (14). Surprisingly, the oldest workers were not more preoccupied by being contaminated than the others, even though they do have a higher risk of severe forms of COVID if contaminated (12). Fear of death seemed shared by all classes of age irrespective to the objective risk. Our findings support previous research reporting that little knowledge on the infection was reported as a risk factor for mental distress in health professionals (9). It has also been previously reported that strong negative emotions lead to misperception of objective information such as probabilities (20), and that they substantially influence one's behavior and ability to adapt it, whereas this ability is critical in the health professional context of the current COVID crisis (19).

### Strengths

The systematic retrospective inclusion of all workers contacting the helpline is a strength of our study; our sample is not self-selected as most recent surveys have been. Our sample is comprised of help-seeking subjects, not representative of all hospital workers, and probably more impacted than others from this hospital. Positive emotions linked to accomplishment were then quasi absent, contrary to previous findings (5), and a quarter of included workers were exhausted, which could lead to burnout states. Moreover, we included any worker's categories, including people exposed to the situation but often suffering from little appreciation, such as cleaning people. In our sample, 16% were not health care providers. We could demonstrate that they endure a significant mental burden too, whereas they are perhaps more vulnerable to psychological risks due to low education level (21). It appears that those poorly visible workers' categories are more psychosocially impacted by the COVID crisis than other more recognized care workers, and should be offered psychological support too.

### Limits

The use of clinical judgment to quantitatively assess mental distress and the lack of a validated scale are limitations of this study. The sample was composed of all contactors in the help service. It is then exhaustive, even if limited in size, since a limited number of professionals contacted the help service. It is not self-reported in the meaning that all contactors were included in the study, i.e., all those who felt the need to contact the helpline. We do not have information on workers who might have sought help elsewhere. All workers except one were women, which is indeed another limit of our study, but in line with previous studies indicating that female health care providers were more heavily impacted than males by the crisis. No medical doctor contacted the helpline and they could have had more difficulty in seeking help, or on the contrary could have been less psychologically impacted than non-medical professionals, as reported in previous studies (22, 23). If our computerized analysis limited *a priori* inclusion of authors in the analysis, our data do include the subjectivity of helpers who wrote the summary with a clinical perspective. Finally, the Occitanie Region, where Nîmes hospital is located, has to date not been overwhelmed by the health crisis, and has been little exposed to ethical questions linked to saturation of the capacity of intensive care units.

### Perspectives

These findings give suggestions for institutions to avoid additional stressors for hospital workers, including both health care providers and logistic/administrative workers. Relying as much as possible on volunteers to organize reassignments, explaining the rapid changes in hygienic instructions and protocols, and considering irrational fears and the individual psychosocial context of each person seem to be crucial keys to be used in crisis management, to limit the psychological impact of the current health crisis.

## Data Availability Statement

The raw data supporting the conclusions of this article will be made available by the authors, without undue reservation.

## Ethics Statement

The studies involving human participants were reviewed and approved by the Center Hospitalier Universitaire de Nîmes local Institutional Review Board. Written informed consent for participation was not required for this study in accordance with the national legislation and the institutional requirements.

## Author Contributions

AL, AC, JM, MB, and GF were responsible for the study concept and design, analysis, and interpretation of data. AL was responsible for statistical analysis and writing of the manuscript. HP contributed to the study concept and design. PP contributed to interpretation of data and review of the manuscript. All authors had full access to all data in the study, take responsibility for the integrity of the data and the accuracy of the data analysis, and have approved the final article.

## Conflict of Interest

The authors declare that the research was conducted in the absence of any commercial or financial relationships that could be construed as a potential conflict of interest.
